# Mechanobiology and the extracellular matrix in pulmonary fibrosis

**DOI:** 10.1016/j.isci.2025.113993

**Published:** 2025-11-11

**Authors:** Ingo Ganzleben, Benjamin D. Medoff

**Affiliations:** 1 Division of Pulmonary and Critical Care Medicine, Massachusetts General Hospital, Boston, MA, USA; 2 Center for Immunology and Inflammatory Diseases, Massachusetts General Hospital, Boston, MA, USA; 3 Harvard Medical School, Boston, MA, USA

**Keywords:** mechanobiology, molecular physiology, cell biology

## Abstract

The extracellular matrix (ECM) is a crucial component of the lung, providing both tissue stability and elasticity. While certain pathologic changes in the ECM, such as fibrotic foci, have long been recognized as hallmarks of pulmonary fibrosis, the matrix has only recently been recognized as an important factor in precipitating fibrosis inception and progression rather than just being an endpoint of fibrotic disease. In this review, we summarize the concept of the ECM as an active player in fibrogenesis, new insights into its qualitative and quantitative composition in pulmonary fibrosis, as well as how cells sense physical matrix properties. Beyond that, we give an overview of cutting-edge technical approaches to study matrix and cell mechano-biology, and, lastly, we summarize current advances in developing mechano-therapeutics to treat fibrotic diseases.

## Graphical abstract

**Figure:**
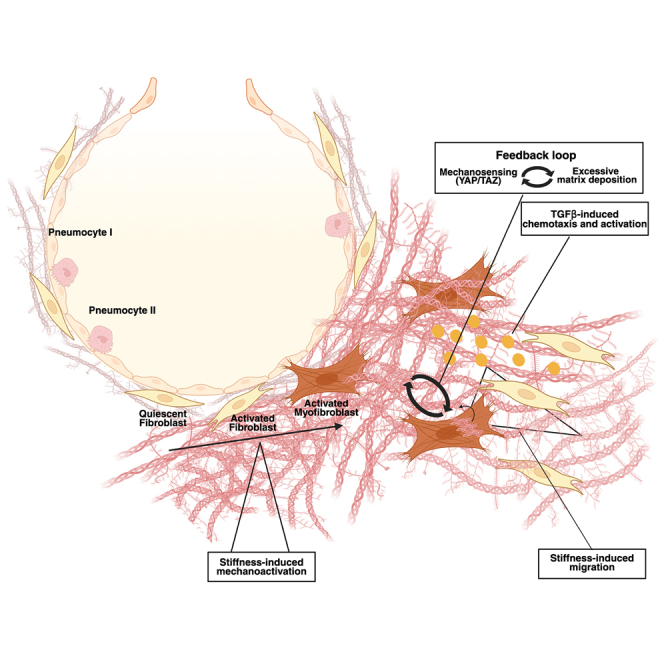

## Pulmonary fibrosis—A disease of the extracellular matrix

Idiopathic pulmonary fibrosis (IPF) is a prototypical fibrotic interstitial lung disease of unknown origin with an incidence of around 1 per 10,000 persons.^1^ IPF is a spatially heterogeneous disease with a typical pathology called usual interstitial pneumonia (UIP), evident on lung histology.^2^ The UIP pattern is reflected by predominantly subpleural and basilar fibrotic changes with severely fibrotic tissue alternating with less fibrotic and even normal-appearing areas of tissue in close proximity to each other. Despite the successful approval of two anti-fibrotic therapies about 10 years ago, pirfenidone and nintedanib,^3^ mortality from IPF remains stubbornly high, with one meta-analysis finding only slight improvement in the cumulative three-year survival rates from the pre-antifibrotic era (61.8%–67.4%) in studies including anti-fibrotic treatment.^4^ This persistent and urgent need to find new therapeutic approaches has sparked intense research that has led to significant advances in the fundamental understanding of the disease. The current paradigm characterizes IPF as a response to chronic epithelial damage, which leads, in genetically susceptible individuals, to epithelial dysfunction and pathologically dysregulated repair processes.^5^^,^^6^^,^^7^^,^^8^ These processes involve a multitude of cells, including but not limited to epithelial cells, endothelial cells, immune cells, and fibroblasts.^9^^,^^10^ Over time, IPF leads to a pathological reorganization and reconfiguration of the extracellular matrix (ECM) with excessive deposition of collagen and other matrix components. This ultimately causes an altered and impaired tissue structure and function, resulting in hypoxia and organ failure. In recent years, there has been a growing appreciation that the ECM is not just a reflection of end-stage organ damage but also is a biologically active player in the inception and progression of fibrosis.^11^^,^^12^ Importantly, while some of these alterations might be specific to IPF, a better understanding of shared common profibrotic mechanisms and pathways involving the physical properties of ECM might lead to the development of more broadly effective anti-fibrotic therapies directly targeting fibrotic organ damage independently of the underlying disease.^13^^,^^14^^,^^15^

## Increased ECM stiffness in lung fibrosis

The ECM consists of a multitude of components, including, but not limited to, collagens, glycoproteins, and proteoglycans. In its entirety, it constitutes the universal scaffold that gives all tissues and organs their form, ensuring proper organ architecture and function.^16^ In the lung, the composition and form of the ECM needs to strike a balance between stability (mainly provided by collagens) and elastic recoil (mainly provided by elastic fibers) to allow for the act of breathing and adequate gas exchange. However, during pulmonary fibrosis, lung stiffness increases with the aberrant deposition of collagen and other ECM components. Booth and colleagues characterized the stiffness of normal lungs and lungs explanted from IPF patients undergoing lung transplant, using atomic force microscopy (AFM), a method to quantify tissue stiffness and viscoelastic properties expressed as Young’s modulus in Pascal [Pa].^17^^,^^18^^,^^19^ They found that the average stiffness in IPF lungs was significantly elevated compared with normal controls. While normal lung tissue had an elastic Young’s modulus of around 1.96 kPa (kPa), IPF tissue was measured at around 16.52 kPa. Decellularization of these lungs revealed several important findings. While normal lung tissue stiffness remained virtually unchanged (1.96 kPa vs. 1.6 kPa), stiffness of decellularized IPF lungs was relatively reduced, while still significantly elevated over control (16.52 kPa vs. 7.34 kPa). These data demonstrate that although the ECM is responsible for the majority of the increased stiffness in fibrotic lung disease, the cellular compartment also contributes to stiffness by contracting the matrix. Besides characterizing the matrix as the relevant substrate of increased organ stiffness, these findings also implied an important role for contractile myofibroblasts in IPF matrix remodeling. Strikingly, recellularizing these IPF ECM scaffolds with normal human lung fibroblasts led to a transforming growth factor β (TGFβ)-independent increase of myofibroblast differentiation and alpha-smooth muscle actin (αSMA) expression, implicating the ECM in facilitating fibrogenesis by directly influencing the biological behavior of fibroblasts.

### Spatial stiffness mapping reveals heterogeneous stiffness distribution in health and disease

To better understand the potential role of ECM stiffness in IPF, it is important to first appreciate that stiffness is not evenly distributed across the lung in either health or disease. AFM studies have shown that the range of Young’s modulus values measured in healthy lungs ranges from 1–10 kPa,^17^^,^^20^ with the parenchyma on the lower end of this spectrum (<3 kPa) compared to blood vessels (∼5 kPa) and airways (∼15 kPa). Importantly, while all these pulmonary structures tend to become somewhat stiffer with age,^20^ fibrotic lungs are, on average, significantly stiffer than healthy lungs, with severely fibrotic areas reaching Young’s modulus values of up to 50 kPa.^17^^,^^21^ AFM microindentation has successfully been used to create topographic stiffness maps in lung fibrosis to better appreciate and characterize the heterogeneous distribution of stiffness in fibrotic lungs overall, and also specifically within fibrotic regions of these lungs.^22^ These findings not only suggest that profibrotic processes are focally increased in the lung (fibroblastic foci) but also that the observed increases in stiffness might be focal catalysts for fibrosis inception and progression.

### Fibroblastic foci as the focus point of matrix remodeling

A hallmark histopathological feature of the UIP pattern in IPF is the presence of fibroblastic foci.^23^^,^^24^^,^^25^ Fibroblastic foci are distinct three-dimensional entities^26^ consisting of activated fibroblasts, contractile myofibroblasts, and excessively secreted ECM. The core of these structures consists of αSMA-positive myofibroblasts associated with collagen I, as well as collagen III, IV, V, VI, fibronectin, hyaluronan, and versican.^27^ These foci are highly active sites of fibrosis inception^28^ and progression^29^ with distinctive transcriptional profiles.^30^^,^^31^ In IPF patients, fibroblastic foci are distinctively more abundant compared to patients with other interstitial lung diseases, and the abundance of fibroblastic foci is correlated with the increased mortality.^32^ However, recent studies using biological pathway analyses in IPF and sarcoidosis have raised the question of whether the pathophysiology of fibroblastic foci is unique to IPF or if it might show similarities across different disease entities at the level of end-stage fibrotic lung disease.^33^

### Molecular mechanisms of fibroblast mechanoactivation

A hallmark publication using an *in vitro* model of hydrogels mimicking pathologically increased stiffness levels typically found in lung fibrosis demonstrated that stiffness-induced myofibroblast activation and collagen expression lead to a distinctive pattern of gene activation. This mechanoactivation acted synergistically with TGFβ-mediated myofibroblast activation and ECM production.^34^ Building on these findings, the authors found that expression of yes-associated protein (YAP) and transcriptional coactivator with PDZ-binding motif (TAZ), as well as their nuclear translocation, was co-localized with areas of increased stiffness in lung tissue from IPF patients.^21^ Beyond this, increased stiffness of the ECM was sufficient to induce nuclear translocation of YAP/TAZ in fibroblasts, leading to myofibroblast differentiation and activation, as well as increased matrix production.^21^ Using knockdown studies, YAP and TAZ were found to be essential for the mechanoactivation of fibroblasts on stiff matrices. Importantly, soft matrices of physiological stiffness (∼1 kPa) conversely had the capability to deactivate IPF fibroblasts, reducing their increased contractility and proliferative rate compared to normal fibroblasts.^35^ A multitude of molecules involved in mechanosensing, such as Rho/Rho-associated coiled-coil kinase (ROCK) and Piezo 1/2, whose detailed functioning goes beyond the focus of this review, have been reviewed in depth elsewhere.^36^ Complementary to these results, fibroblasts have been demonstrated to assume the stiffness of the matrix they sit upon, e.g., mediated by crosslinking of filamentous actin (F-actin) filaments.^37^ This observation opens the possibility that the cellular compartment itself manifests increased stiffness in IPF. This hypothesis is supported by the finding that fibroblasts from fibrotic lungs are stiffer than controls and show a larger increase in stiffness in response to TGFβ.^38^^,^^39^

## Qualitative and quantitative composition of the ECM in IPF

The ECM of the lung provides structural scaffolding for the organ. At the same time, the physical properties of the matrix need to allow for dynamic expansion upon inspiration and sufficient elastic recoil upon expiration. This delicate balance is based on an ongoing turnover of ECM components and bidirectional interactions between the acellular ECM and the cellular compartment.^40^^,^^41^^,^^42^ As a structural correlate to the different stiffness levels observed in distinct compartments of the lung, the local composition of the ECM differs based on the specific (mechanical) demands. For example, alveoli need to elastically change their geometry with respiration,^43^^,^^44^ conducting airways provide stability with a more rigid ECM rich in collagen,^45^^,^^46^ and the vasculature is adapted to the pressure demands of blood vessels of different sizes.^47^

### The matrisome—A systematic approach to understanding the ECM

A systematic approach to understand the composition of the ECM has been undertaken by Alexandra Naba and colleagues using proteomics.^48^ The core matrisome (Table 1) for *Homo sapiens* encompasses 275 proteins subdivided into 44 collagens, 195 glycoproteins (e.g., elastin, fibulins, fibrillins, fibronectin, and laminins), and 35 proteoglycans (e.g., decorin, lumican, and versican). Larger than the actual core matrisome itself is the group of 753 matrisome-associated proteins encompassing 171 ECM-affiliated proteins (e.g., annexins), 238 ECM regulators (e.g., matrix metalloproteinases and lysyl oxidases), and 344 secreted factors (e.g., TGFβ, Bone Morphogenetic Proteins [BMPs]).^48^

**Table 1:** Overview of the matrisome as defined by Naba et al.

Core matrisome (274)	Matrisome-associated (753)
44 Collagens	171 ECM-affiliated Proteins
195 ECM Glycoproteins	238 ECM Regulators
35 Proteoglycans	344 Secreted factors

Since its inception, the concept of the matrisome has evolved rapidly,^49^ and as of 2023, a searchable database of matrisomal gene expression is available for *Homo sapiens* and *Mus musculus*,^50^ with a multitude of regulators of the matrisome having been identified.^51^^,^^52^ Several studies have characterized aging-related matrisomal changes in model organisms such as *Caenorhabditis elegans*,^53^ mice,^54^^,^^55^ and humans.^56^^,^^57^^,^^58^

Interestingly, the core matrisome of the human lung also shows distinctive sex-specific changes with increasing age, with both up- and down-regulation of matrisome components.^59^ Using a multi-omics approach, an age-related upregulation of a set of ECM proteins (COL1A1, COL6A1, COL6A2, COL14A1, FBLN2 [fibulin-2], LTBP4 [latent TGFβ-binding protein 4], and LUM [lumican]) was recently identified in the lung in general, with a compartment-specific upregulation of COL1A1 in the lung parenchyma.^60^ These findings might help to explain the predilection of IPF for older and male patients. Several other studies also support the notion of sex-based differences in pulmonary fibrogenesis. Elizabeth Redente and colleagues demonstrated that male mice develop a greater increase in lung stiffness compared to female mice in the bleomycin model of lung fibrosis.^61^ Another elegant study exposed human pulmonary arterial adventitial fibroblasts (hPAAFs) to sera from male or female individuals of different ages and demonstrated that mechanosensing of stiff matrix with subsequent activation occurred independently of sex or age in male hPAAFs. In contrast, female hPAAFs required high serum estradiol levels to show the same biological behavior.^62^ This is particularly intriguing as the epidemiology of pulmonary arterial hypertension shows a clear female predilection.^63^ It also seems that these sex-specific differences are not limited to pathologies of the lung but rather represent more profound aspects of fibroblast biology, as studies focusing on fibrocalcific aortic valve disease have shown.^64^^,^^65^^,^^66^

### Disease-specific matrisomal phenotypes

Building on these advances, matrisomal profiling has identified specific “matritypes” in several lung diseases^67^^,^^68^^,^^69^ and multiorgan fibrosis.^70^ Surprisingly, it was revealed that almost all differentially regulated matrisomal proteins in end-stage cystic fibrosis were actually downregulated, while the overall abundance of the matrix was not changed compared to control lungs. Hence, the main pathology was unexpectedly based on matrix dysregulation and pathological reorganization with a less diverse composition of the ECM rather than on increased matrix deposition.^67^ Recent studies have used compartment-specific decellularization to characterize disease- and region-specific changes in IPF and chronic obstructive pulmonary disease (COPD), with differences found in glycosaminoglycan composition.^71^^,^^72^ Another comparative analysis of patients with end-stage lung disease showed distinctive matrisomal phenotypes for end-stage COPD compared with IPF, which had decreased cell-adhesion mediating laminins (laminin subunit beta-1 [LAMB1] and laminin subunit gamma-1 [LAMC1]) and increased collagen VI, suggesting altered adhesion between cells and the ECM as an important aspect of the underlying pathophysiology of IPF.^68^ Fittingly, a recent study observed that changes in the matrix deposited by IPF lung fibroblasts, in particular, a specific overexpression of the ECM glycoprotein secreted protein acidic and rich in cysteine, caused impaired cell migration of keratin 5-positive basaloid cells through the three-dimensional cell-derived matrix.^73^ On the other hand, the more aligned collagen fibers in ECM scaffolds modeled after the matrix pattern of IPF lungs have been implicated in leading to a higher migration speed of fibroblasts.^74^ A larger study generated a more detailed profile of the matrisomal phenotype of IPF, showing 56 differentially regulated proteins with both up- (19 proteins) and down- regulation (37 proteins) being observed.^69^ Based on their explorative approach, the authors also identified several proteins with unknown functions that warrant further study (Table 2). In general, decellularized matrix derived from IPF lung tissue shows a distinctive matrisomal protein profile with a typical upregulation and downregulation of certain groups of matrisomal proteins. Upregulated in the context of lung fibrosis are several collagens and proteoglycans, such as decorin,^75^ lumican,^76^^,^^77^ versican,^78^ and glycosaminoglycans.^79^ There also seems to be a profound dysregulation of the basement membrane, both with regard to its quantitative composition and structure.^80^^,^^81^^,^^82^^,^^83^^,^^84^ Importantly, healthy fibroblasts seeded onto IPF-derived ECM scaffolds were reprogrammed and produced an altered matrix composition similar to what they encountered, with a particular focus on basement membrane proteins and periostin.^80^ This suggests that the pathologically changed matrix in IPF perpetuates itself by reprogramming cell behavior.^85^ Recent findings also implicate compositional changes of the ECM that potentially precede stiffness changes in more acute settings such as acute respiratory distress syndrome (ARDS)^86^ and COVID-19,^87^ the latter linking the extent of matrix turnover to patient prognosis. This subject has also been intensively reviewed by Janette Burgess and colleagues.^88^^,^^89^

**Table 2:** Overview of matrisome phenotype examples

Disease	Matrisome findings	Study
COPD, IPF	COPD • Increased matrix metalloproteinase 28 (MMP28) • Increased metalloproteinase inhibitor 3 (TIMP3) IPF • Decreased cell-adhesion mediating laminins ○ Laminin subunit beta-1 (LAMB1) ○ Laminin subunit gamma-1 (LAMC1) Increased collagen VI	Åhrman et al.^68^
IPF	Increased secreted protein acidic and rich in Cysteine (SPARC)	Hewitt et al.^73^
IPF	56 differentially regulated proteins in IPF (19 upregulation, 37 downregulation, examples) • Collagen, type I, alpha 1 (COL1A1) • Secretoglobin Family 1A Member 1 (SCGB1A1) • Transgelin (TAGLN) • Presenilin 2 (PSEN2) • Tetraspanin1 (TSPAN1) • Cathepsin B (CTSB) • Anterior Gradient 2 (AGR2) • Versican (CSPG2) • Serpin family B member 3 (SERPINB3) Novel proteins with unknown function • Galectin-7 (LGALS7) • Asporin (ASPN) • Heat shock protein 90 alpha family class A member 1 (HSP90AA1) Heat shock protein 90 alpha family class B member 1 (HSP90AB1)	Tian et al.^69^
IPF	Reduction collagen XIV	Nizamoglu et al.^90^

### Core matrisome—Collagens

Several core matrisome family members are dysregulated in IPF, with fibrillar collagens (I, II, III, V, XI, XXIV, and XXVII)^91^ playing a particularly important role. Fibrillar collagens are among the most abundant proteins in the lung and a main determinant of tensile tissue strength.^92^ Baseline levels of collagen I, III, as well as the non-fibrillar collagen VI have been shown to be increased in patients with IPF compared with healthy controls. Beyond that, their increased turnover has been indicative of progressive disease.^93^^,^^94^^,^^95^ Collagen XIV, a member of the fibril-associated collagens with interrupted triple helices, supports the regulation of collagen fibril formation, deposition, and maturation in a process called fibrillogenesis,^96^ thereby making an important contribution to the regulation of the overall ECM structure.^97^^,^^98^ Interestingly, collagen XIV has recently been found to be reduced in the lung tissue of patients with IPF.^90^ This substantiates that, in addition to increased collagen deposition, pathologically reduced levels of certain collagens can contribute to the deposition of a disorganized fibrotic ECM, leading to pulmonary fibrosis with fibrotic tissue damage and disease progression.^99^ Similarly, collagen IV, an important part of the basement membrane,^100^ was downregulated in IPF, particularly in fibrotic foci.^29^ Intriguingly, matrix metalloproteinase 2 (MMP2)-dependent degradation of basement membrane collagen IV has been described as a consequence of ROCK-mediated α6-integrin overexpression in IPF fibroblasts in response to increased matrix stiffness. This, in consequence, leads to an invasive myofibroblast phenotype that is necessary for pulmonary fibrogenesis.^101^ Complementarily, increased TGFβ-mediated deposition of structurally abnormal α1 and α2 chains of collagen IV has been observed in IPF.^102^^,^^103^^,^^104^ It has been speculated that those deposits in early fibrotic lesions impair the outward migration of fibroblasts by reducing their mobility, “trapping” activated fibroblasts once they have arrived, thereby facilitating increasing fibrosis.^102^ Laminins, other essential components of the basement membrane, have likewise been shown to be involved in multiple profibrotic processes, such as ECM production and myofibroblast deposition.^105^ For an in-depth review on the role of different collagens in lung fibrosis, we want to highlight several recently published reviews on the subject.^90^^,^^106^^,^^107^^,^^108^^,^^109^

### Core matrisome—Elastic fibers as a nexus of ECM reorganization in IPF pathogenesis

Elastic fibers are highly complex structures that give lung tissue the elastic properties necessary for the dynamic recoil underlying inflation and deflation during breathing.^110^ The biosynthesis of elastic fibers, called elastogenesis, starts with tropoelastin monomers being deposited on the outer side of the cell membrane of the elastin-producing cell.^111^^,^^112^ Subsequently, tropoelastin monomers first coalesce into nanoparticles that remain attached to the cell surface. These nanoparticles then form spherules of 1–2 μm size until they are finally deposited onto scaffolds (microfibrils) made of fibrillin molecules. This maturation process of elastic fibers involves several players, including lysyl oxidase (LOX) and lysyl oxidase-like (LOXL) crosslinking enzymes, as well as fibulins. In particular, fibulin-4 and fibulin-5 are binding partners for LOXL1 and are essential for the integrity and proper function of mature elastic fibers.^113^^,^^114^ It has long been known that elastin gene expression is increased in pulmonary fibrosis,^115^ and more recently, it has been shown that increased levels of elastic fibers in the lung are associated with worse outcomes in patients with IPF.^116^ On the other hand, degradation of elastic fibers also plays an important biological role in fibrotic lung disease. A recent study available as a preprint indicated that the serum amount of “elastokines,” which describe elastin degradation products, is negatively correlated with both the forced vital capacity and three-year transplant-free survival.^117^ The authors speculated that these elastin-degradation products reflect an increased pathological turnover of the matrix.

### Core matrisome—Fibulins are ECM glycoproteins at the intersection of TGFβ signaling and mechanoactivation

Fibulins, secreted glycoproteins of the ECM,^118^ are involved in pathologically dysregulated remodeling of the lung.^119^ In general, fibulin-1 is increased in IPF, correlates with disease progression,^120^ and plays a role in pulmonary fibrogenesis *in vivo.*^121^ On a functional level, fibulin-1c seems to activate latent TGFβ by interacting with the LTBP1, thereby facilitating myofibroblast activation and ECM deposition via increased mothers against decapentaplegic homolog 3 (SMAD3) signaling.^121^ Other observations imply a role in modulating cell motility in a cell-specific fashion and increasing fibroblast attachment.^122^^,^^123^ Similarly, fibulin-2 has only recently been recognized as a potentially important mediator of TGFβ-induced fibroblast migration via focal adhesion kinase (FAK) and fibroblast activation^124^ while also being important for basement membrane integrity.^125^^,^^126^ In contrast, fibulin-5 seems to perpetuate fibrosis, at least in part, independently of TGFβ by increasing tissue stiffness,^127^ with results from asthma patients suggesting a fibulin-5-mediated modulation of the YAP/TAZ and Hippo pathway based on β1 integrin binding in airway smooth muscle cells.^128^ Fibulin-5 deposition during elastogenesis, in turn, depends on binding LTBP 4, which also seems to be independent of TGFβ function.^129^^,^^130^ Still, there is also evidence of a more direct interaction between TGFβ and fibulin-5^131^^,^^132^^,^^133^^,^^134^^,^^135^ (Table 3).

**Table 3:** Overview of relevant proteins and their functions in IPF

Protein	Functions	Study
CCN1 Cysteine-rich protein 61 (CYR61)	• Regulated by YAP • Pro- and anti-fibrotic effects • Role in controlling cellular senescence and senescence-associated secretory phenotype (SASP) • Activates FAK signaling • Enhances TGFβ/SMAD3 signaling in fibroblasts • CCN1 plasma levels negatively correlate with transplant-free survival in IPF • Controls matrix adhesion and cellular migration in skin fibroblasts	Totaro et al.^136^; Zhu et al.^137^; Kurundkar et al.^138^ Kerek et al.^139^ Wu et al.^140^ Kulkarni et al.^141^ Grzeszkiewicz et al.^142^ Tsou et al.^143^; Borkham-Kamphorst et al.^144^
CCN2 Connective Tissue Growth Factor (CTGF)	• Regulated by YAP • Strong profibrotic mediator, anti-fibrotic in some contexts associated with SASP • Facilitates FAK-PI3K-Akt signaling via binding to fibronectin, integrin α5β1 • Important role in cell adhesion and migration • Importance for sustaining fibrotic response • CCN2 directly facilitates TGFβ1 signaling • Therapeutic CCN2 inhibition (FG-3019/pamrevlumab) ○ Promising results in preclinical models of organ fibrosis ○ Promising results in phase 2 study in IPF patients ○ Failed in phase 3 clinical trial in IPF patients (no improvement in primary outcome (absolute change in FVC) after 48 weeks • Recent work explores topical application of anti-CCN2 Anticalin protein PRS-220	Totaro et al.^136^; Chen et al.^145^ Abreu et al.^146^ Parapuram et al.^147^ Makino et al.^148^; Yanagihara et al.^149^; Tam et al.^150^ Richeldi et al.^151^; Leask^152^ Mori et al.^153^; Jun and Lau^154^; Raghu et al.^155^ Neiens et al.^156^
CCN3 Nephroblastoma Overexpressed (NOV)	• Mostly antifibrotic mediator	Yin et al.^157^
CCN4 WNT1-inducible-signaling pathway protein 1 (WISP-1)	• Regulated by YAP • Downstream target of TGFβ • Profibrotic effect in murine lung fibrosis model • Increased in alveolar epithelial type II (ATII) cells in murine lung fibrosis and human IPF lung fibroblasts • Facilitates proliferation and EMT in primary murine ATII cells • Facilitates increased ECM production in human and murine lung fibroblasts	Totaro et al.^136^; Königshoff et al.^158^^,^; Singh et al.^159^
CCN5 WNT1-inducible-signaling pathway protein 2 (WISP-2)	• Negative regulator of fibrosis progression in endometriosis via Wnt/β-catenin/SMAD3 signaling	Liu et al.^160^
Decorin	• Antifibrotic • Serum levels are a potential prognostic marker in IPF • Regulates TGFβ bioavailability and pathway activation (pSMAD2) • Tissue protective regulation of MMPs • TGFβ downregulates decorin	Markmann et al.^161^; Ferdous et al.^162^ Ohto-Fujita et al.^163^; Al Haj Zen et al.^164^; Kähäri et al.^165^; Li and Velleman^166^; Kolb et al.^167^; Kolb et al.^168^; Nikaido et al.^169^
Fibulin-1	• Increased in IPF • Correlates with disease progression • Activates latent TGFβ • Regulation of cell motility • Regulation of fibroblast attachment	Jaffar et al.^120^; Liu et al.^121^
Fibulin-2	• Regulates TGFβ-induced fibroblast migration and activation via FAK • Function in basement membrane integrity	Zhang et al.^124^; Ibrahim et al.^125^; Longmate et al.^126^
Fibulin-5	• Increases tissue stiffness • Modulation (via specific β1 integrin binding) ○ YAP/TAZ activation ○ Hippo pathway activation • Deposition linked to LTBP4 binding • Direct interaction with TGFβ	Nakasaki et al.^127^; Fu et al.^128^ Noda et al.^129^; Dabovic et al.^130^; Lee et al.^131^; Topalovski et al.^132^; Kuang et al.^133^; Schiemann et al.^134^; Tsuda^135^
Lumican	• Profibrotic • Binds TGFβ type I receptor (TBR1) → phosphorylation of ERK1/2, SMAD2/3 • Elevated during early fibrotic response in ARDS • Directly increased by TNFα • Mediates stretch-augmented EMT (VILI) via ERK1/2 • Increased fibroblast contractility (integrin α2)	Pilling et al.^76^; Wang et al.^77^; Yamanaka et al.^170^; Xiao et al.^171^; Li et al.^172^ Liu et al.^173^; Engebretsen et al.^174^
Periostin	• Profibrotic • Multi pathway modulator ○ JAK ○ MAPK ○ NF-κB ○ PI3K/Akt ○ RhoA/ROCK ○ Wnt ○ TGFβ • Involved in fibroblast cell cycle regulation (via integrin α_V_β_3_) • TGFβ signaling augmentation (via integrin α_V_β_3_/β_5_-SMAD3)	Wang et al.^175^; Naik et al.^176^; Okamoto et al.^177^; Yoshihara et al.^178^; Tirunavalli et al.^179^

IPF, idiopathic pulmonary fibrosis; TGFβ, transforming growth factor beta; SASP, senescence-associated secretory phenotype; FAK, focal adhesion kinase; pSMAD2, phosphorylated Mothers against decapentaplegic homolog 2; YAP/TAZ, yes-associated protein 1/transcriptional co-activator with PDZ-binding motif; MMPs, matrix metalloproteinase; LTBP 4, latent transforming growth factor beta-binding protein 4; ERK 1/2, extracellular signal-regulated kinases (ERKs); EMT, epithelial-mesenchymal transition (EMT); ARDS, acute respiratory distress syndrome; TNFα, tumor necrosis factor α; VILI, ventilator-induced lung injury; JAK, Janus kinase; MAPK, mitogen-activated protein kinase; NF-κB, nuclear factor kappa B; PI3K, phosphatidylinositol 3-kinase; Akt, protein kinase B; RhoA, Ras homolog family member A; ROCK, Rho-associated protein kinase; Wnt, Wingless-related integration site.

### Core matrisome—ECM glycoproteins and proteoglycans have versatile functions in TGFβ signaling and mechanoactivation

The ECM proteoglycan decorin acts as a regulator of matrix homeostasis by modulating TGFβ function and, in particular, its bioavailability.^161^^,^^162^ Decorin is capable of both reducing TGFβ activity by sequestering it through direct binding and by downregulating TGFβ pathway activation via reduced TAZ and pSMAD2 translocation into the nucleus.^163^ It also plays a role in preserving tissue integrity during matrix remodeling by downregulating MMP-1 and MMP-3 while upregulating MMP-2 and TIMP-2.^164^ Interestingly, TGFβ itself downregulates decorin expression.^165^^,^^166^ Accordingly, overexpression of decorin in murine disease models was sufficient to protect mice from TGFβ overexpression-induced lung fibrosis.^167^^,^^168^ More recent evidence has suggested that serum levels of decorin could be used as a prognostic marker in patients with IPF.^169^ In contrast, core matrisome proteoglycan lumican is profibrotic by binding the TGFβ type I receptor, inducing phosphorylation of ERK1/2 and SMAD2/3.^170^^,^^171^ It was also elevated during the early fibrotic response in patients with lung injury and ARDS and in experimental ARDS.^77^ Interestingly, lumican was directly increased by TNFα,^76^ and it directly mediated stretch-augmented epithelial-mesenchymal transition in a ventilation-induced lung injury model via activation of ERK1/2.^172^ There is further evidence of lumican-mediated increased fibroblast contractility through the binding of integrin α2.^173^ Overall, it seems that lumican upregulation occurs in response to multiple inflammatory and mechanical stimuli, particularly stretch.^174^ Other matrisomal proteins that are upregulated in IPF include tenascin-C^180^ and versican, which can be found in the vicinity of myofibroblasts in early fibrotic foci in IPF but do not seem to be specific to IPF, but more broadly involved in fibrotic pulmonary diseases^78^^,^^181^ by regulating inflammatory processes.^182^^,^^183^ Versican accumulation due to decreased turnover is also part of the distinctive ECM remodeling profile observed during acute exacerbation of IPF and is associated with increased mortality.^184^ Periostin, a core matrisome ECM glycoprotein, interacts with a multitude of pathways, including JAK, MAPK, NF-κB, PI3K/Akt, RhoA/ROCK, Wnt, and TGFβ.^175^ Based on this, it is no surprise that this profibrotic mediator^176^^,^^177^ has been ascribed diverse functions, including fibroblast cell cycle regulation by binding to integrin α_V_β_3_.^178^ Beyond this, periostin has been characterized as a central mediator in pulmonary fibrogenesis by augmenting TGFβ signaling^179^ via integrin α_V_β_3_/β_5_-SMAD3 integrative crosstalk signaling that could be therapeutically exploited to ameliorate lung fibrosis in murine disease models.^185^

The “cellular communication network factor” (CCN) protein family (Table 3) represents another crucial group of core matrisome ECM glycoproteins that play an important role in the development of fibrotic lung disease, as reviewed by Sun and colleagues.^186^ CCN1 (CYR61), CCN2 (CTGF), and CCN4 (WISP1) are regulated by YAP signaling, connecting the matrix mechanosensing of increased stiffness to the upregulation of pro-fibrotic mediators in the form of a feedforward loop.^136^

CCN1, also known as cysteine-rich protein 61 (CYR61), has been characterized as an important profibrotic mediator in lung injury^137^ by enhancing TGFβ/SMAD3 signaling in fibroblasts.^138^ Even more, in a recent study, plasma levels of CCN1 were negatively correlated with transplant-free survival in a large cohort of IPF patients,^141^ stressing its potential as a prospective target in treating the development and progression of fibrotic lung disease. Mechanistically, binding of CCN1 to specific integrins seems to be crucial to its function. For example, its binding to integrin αvβ5 has been shown to control matrix adhesion and cellular migration in skin fibroblasts.^142^ There is also further direct evidence that CCN1 binding to integrins α6β1 and ανβ3 leads to increased FAK signaling,^140^ which in turn increases nuclear YAP translocation, closing the feedforward loop of YAP-CCN1-YAP-mediated myofibroblast activation. The complex role of CCN1 in controlling cellular senescence and the senescence-associated secretory phenotype has been reviewed elsewhere^139^ and is beyond the scope of our current review.

CCN2, also known as connective tissue growth factor (CTGF), is a strong pro-fibrotic mediator, but, similar to CCN1, it seems to have antifibrotic effects under certain circumstances, mainly by association with an antifibrotic senescence-associated secretory phenotype in fibroblasts.^143^^,^^144^^,^^153^^,^^154^ Functionally, CCN2 is involved in binding fibronectin, integrin α5β1, and others to facilitate FAK-PI3K-Akt signaling,^145^ enabling fibroblast matrix interactions, including adhesion and migration. Interestingly, disinhibition of the PI3K-Akt signaling pathway due to reduced “phosphatase and tensin homolog (PTEN)” in lung fibrosis seems to be dependent on CCN2 function.^147^ Adding another layer of complexity, CCN2 in turn also directly facilitates TGFβ1 signaling, a direct negative regulator of PTEN.^146^ Therapeutic inhibition of CCN2 through the monoclonal antibody FG-3019/pamrevlumab yielded promising results in preclinical models of organ fibrosis^148^^,^^149^^,^^150^ as well as promising results in a phase 2 study in IPF patients.^151^^,^^152^ Unfortunately, a phase 3 clinical trial in IPF patients failed, showing no improvement in the primary outcome (absolute change in forced vital capacity [FVC]) after 48 weeks.^155^ The reason for this failure is still unknown and might be due to redundant functionality within the CCN protein family. However, a recent study has speculated that systemic drug application, as used so far, might generate insufficient tissue levels of the antibody. In a recently published study, the investigators generated a specific anti-CCN2 Anticalin protein (PRS-220) and performed a detailed characterization of its pulmonary delivery via oropharyngeal aspiration, including *ex vivo* ventilation-perfusion models of human lungs and an *in silico* inhalation study in three human subjects. The pulmonary deposition in fibrotic lung tissue achieved by inhalation of PRS-220 was also superior to that achieved by systemic application of FG-3019/pamrevlumab in a murine disease model. Importantly, increased pulmonary deposition of PRS-220 resulted in a significantly stronger reduction of lung fibrosis compared with systemic treatment with FG-3019/pamrevlumab in these mice. Beyond this, the authors also offered a proof-of-principle effectiveness of PRS-220 using a human precision cut lung slice (PCLS) model of IPF.^156^ Overall, this study offers a potential way forward for anti-CCN2 therapies in the treatment of IPF.

### Matrisome-associated proteins regulate matrix configuration and turnover

Matrisome-associated proteins contain ECM regulators that function as modulators of ECM composition and homeostatic remodeling. Prominent examples are the LOX and LOXL enzymes, which, by enzymatically contributing to the maturation of both collagen and elastic fibers, make compounds of the ECM insoluble and thereby contribute to the maturation and stability of the matrix.^187^ LOX and LOXL family members have a vital function in crosslinking both elastic and collagen fibers by, as the name suggests, oxidizing lysyl residues,^188^ thus ensuring proper configuration of the ECM.^189^^,^^190^ Dysregulated LOX activity has been demonstrated to contribute to fibrotic tissue reorganization in systemic sclerosis,^191^ while the increased activity of LOXL1 and LOXL2 has been described in IPF fibroblasts compared to controls. Experiments with these primary human fibroblasts have shown the effectiveness of pan LOX inhibitors in abrogating TGFβ-mediated collagen remodeling.^192^^,^^193^ The relative contributions of the several LOXL enzyme isoforms in IPF, however, are less clear, with a partially redundant function of the isoforms likely. Beyond actual enzymatic collagen crosslinking, LOXL1 plays a role in mediating TGFβ-induced transcriptional changes, such as an increase in αSMA and collagen I expression via SMAD2/3 phosphorylation.^194^ Knocking out LOXL1 was also a successful way of protecting mice from TGFβ-overexpression-induced pulmonary fibrosis *in vivo.*^195^ This versatile functionality of LOXL1 has been speculated to be based on proteomic cleavage of LOXL1 by BMP1 and others^196^^,^^197^ into mature LOXL1 and several other fragments of varying length, with not fully defined functional profiles.^198^ Other studies have also generally implicated LOXL2 and LOXL3 in fibroblast activation in IPF.^199^ While serum levels of LOXL2 have been associated with an increased risk for IPF progression,^200^ recent results have suggested that LOXL4, and not LOXL2, is the essential LOXL isoform regulating pulmonary fibrogenesis.^201^ In IPF, fibroblasts produce an altered ECM that facilitates enhanced fibroblast adhesion and proliferation. Inhibiting LOX and the crosslinking enzyme transglutaminase-2 led to an abrogation of the ultrastructural changes observed in IPF-derived ECM. Reversing these structural changes also neutralized the increased fibroblast proliferation and adhesion. This supports the notion that distinctive changes in the composition and structure of ECM produced by IPF fibroblasts are sufficient to influence fibroblast activation status and behavior.^12^ MMPs, enzymes that remodel the ECM,^202^ have several family members, such as MMP-2 and MMP-9, which have been shown to be elevated in a murine disease model of IPF.^203^ However, their knockout did not prove protective results, suggesting their increase might be a byproduct of increased tissue remodeling. Even more, a recent study suggests a potential antifibrotic function of MMP-2 as its overexpression ameliorated lung fibrosis in the bleomycin model.^204^ This fits the overall understanding of a highly diverse function of different MMPs in the pathogenesis of IPF with both pro- and anti-fibrotic functions.^205^ The overall pro- or anti-fibrotic function depends on the interplay between MMPs and their inhibitors, the tissue inhibitors of metalloproteinases.^206^

### TGFβ, a matrisome-associated protein, is stored in the matrix and activated mechanically

One of the most important members of the “matrisome-associated” protein subgroup of secreted factors is TGFβ. TGFβ is first translated as a pro-peptide containing the mature TGFβ section and the latency-associated peptide (LAP) sequence, which is then cleaved and reassembled non-covalently into the small latent complex (SLC).^207^ An important next step is the binding of LAP to LTBPs, which happens in the form of covalent binding in the endoplasmic reticulum.^208^ LTBPs, themselves core matrisome proteins, have a relevant and interesting homology with the core matrisome proteins fibrillin-1 and fibrillin-2, with the members of this family sharing a unique domain, the 8-cysteine (8-Cys) domain.^209^ Via their LAPs, the TGFβ isoforms bind covalently to LTBP 1, 3, and 4.^210^^,^^211^^,^^212^ While LTBP 1 and LTBP 3 are strongly bound to these LAP domains, LTBP 4 is less effective in doing so and can only bind to TGFβ1. The big outlier in the LTBP family is LTBP 2, which does not bind TGFβ at all^212^ but plays an integral role in maintaining the stability of zonular fibers,^213^ reflecting wider evidence of an independent role of LTBPs 2 and 4 for microfibril structure and stability.^214^ Interestingly, all four LTBP isoforms bind to microfibrils and are structurally related to fibrillin.^215^^,^^216^ The combination of TGFβ, LAP, and the bound LTBP is called the large latent TGFβ complex. This complex is secreted into the ECM, where it binds to fibrillin microfibrils,^217^^,^^218^ fibronectin,^219^ and fibulins^217^ as part of the fibulins’ dual function in regulating TGFβ availability and facilitating structural integrity of elastic fibers.^119^^,^^129^^,^^130^^,^^220^ Liberating TGFβ from this complex, thus activating it, can be achieved by different means.^221^ Thrombospondin 1 binds to LAP to facilitate TGFβ activation by inducing a conformational change.^222^^,^^223^^,^^224^ Fascinatingly, reactive oxygen species (ROS) are also capable of directly inducing a conformational change through oxidation of LAP to activate TGFβ.^225^ More broadly, ROS also act in this context by influencing other enzymes, such as TSP1 and MMPs.^226^^,^^227^ Additionally, increased lactic acid in IPF has been shown to activate latent TGFβ by reducing the pH of the environment.^228^ Proteases are also capable of activating latent TGFβ in IPF.^229^

Particularly interesting from a mechanobiology perspective is the integrin-mediated activation of latent TGFβ via conformational changes^230^ following the “straight-jacket” hypothesis, which has been compared to the unwrapping of a candy.^231^ The SLC complex, consisting of TGFβ and LAP, is anchored via its Cys33 residue to the ECM by binding to the 8-Cys domain of the LTBP, which in turn is connected to the ECM. Integrin α_v_β_6_ was the first integrin described to bind and activate latent TGFβ1,^232^ and now it is understood that integrins α_V_β_1/3/5_^233^ are also relevant in this mechanism. Their expression is a *conditio sine qua non* for the mechanical activation of TGFβ1 and TGFβ3, while TGFβ2 seems to lack the necessary “arginylglycylaspartic acid (RGD)”-binding sequence on the LAP.^231^ The integrins are bound on the cell surface of (myo-)fibroblasts and bind to LAP at this conserved RGD-binding site^234^ to pull open the “straight-jacket” and liberate active TGFβ1. The ECM serves as a counter bearing in this process, making the ECM properties a major determinant of the extent of mechanically mediated TGFβ1 liberation. On the other side of this equation, the contraction of myofibroblasts is in itself sufficient to liberate TGFβ1. However, the extent of this process is also determined by the stiffness of the opposing matrix, with the extent of release increasing with increasing stiffness of the ECM.^235^ In seminal work, it has been shown that increased fibrotic remodeling of the lung ECM creates a pre-strain in the ECM that increases the susceptibility of the tissue to release TGFβ1 after a mechanical strain stimulus.^236^ This finding bears significant implications for understanding the progression of fibrotic diseases, as mechanical stress in itself might perpetuate and propagate fibrosis via this mechanism,^237^ which in IPF includes any form of mechanical ventilation.^238^^,^^239^^,^^240^

## Mechanosensing and the ECM

### A closer look at the physical dimensions of stiffness

Tissues and their ECM behave as viscoelastic objects. This means that tissues have properties of both elastic and viscous objects. Young’s modulus (or storage modulus) reflects the energy conserved in an elastic object when a force is applied. This energy is stored and released once the force is no longer present. It is complemented by the loss modulus, which refers to the energy dissipated in the force-induced deformation of a material or tissue with viscous properties.^241^ Viscoelastic soft tissues tend to have a loss modulus of about 10%–20% of their storage modulus. When some tissues face a force exceeding a certain limit, they deform constantly, a behavior described as viscoplasticity. Interestingly, most ECM components, collagen, and the matrix of the basement membrane, fall into this category^242^^,^^243^ unless they are strongly cross-linked.^244^

### Fibroblasts sample the stiffness of their environment in a dynamic process

**Figure 1:**
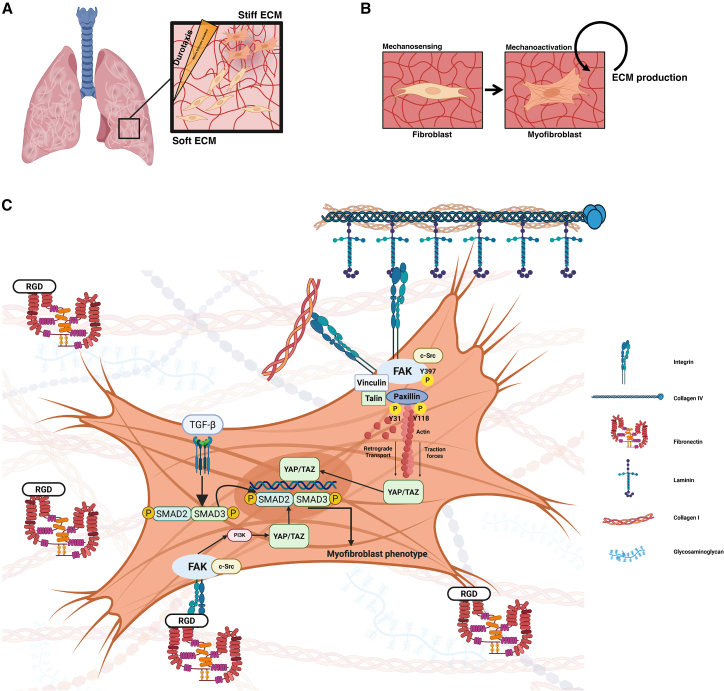
Increased matrix stiffness drives lung fibrosis through fibroblast mechanoactivation Created in BioRender. Medoff, B. (2026) https://BioRender.com/e5v11sn and https://BioRender.com/m9myarz. (A) Illustrates the heterogeneous distribution of fibrosis in the lung in IPF on the macroscopic level (left). The inset shows an equally heterogeneous distribution on the histological level with areas of relatively unchanged extracellular matrix (“Soft ECM”) adjacent to severely altered fibrotic ECM (“Stiff ECM”) in the form of a fibroblast focus. The resulting matrix stiffness gradient is sensed by fibroblasts, which then migrate along the gradient toward the stiffer matrix and transdifferentiate into myofibroblasts. (B) Illustrates the vicious cycle of stiffness-induced matrix production. Fibroblasts directly sense the increased stiffness of pathologically altered extracellular matrix through mechanosensing and transdifferentiate into myofibroblasts (mechanoactivation). Activated fibroblasts then produce more and stiffer ECM, completing the vicious cycle. (C) Illustrates multiple mechanosensing pathways that converge on the central mechanosensor YAP. Integrin binding to multiple ECM components, such as laminin and collagen (right upper corner) in the context of focal adhesions, activates FAK through phosphorylation on tyrosine 397. FAK then associates with c-Src, vinculin, talin, and paxillin, whose phosphorylation on tyrosine residues 31 and 118 leads to further signal transduction via the actin cytoskeleton. Integrin binding of fibronectin via its RGD domain (left lower corner) activates YAP translocation via the FAK-c-Src-PI3K axis. Synergistically, TGFβ receptor activation leads to, among others, phosphorylation and nuclear translocation of SMAD2/SMAD3, which then translocate into the nucleus and act synergistically with YAP (left upper corner).

Fibroblasts and other cells probe the ECM by using coupling molecules that connect to binding domains in ECM molecules. Integrins are some of these crucial connectors, transducing information about the physical properties of the matrix into the cell^245^ (Figure 1). Integrins bind to collagen (α_1_β_1_, α_2_β_1_, α_10_β_1_, and α_11_β_1_), laminin (α_3_β_1_, α_6_β_1_, α_6_β_4_, and α_7_β_1_), and via the conserved RGD domain to fibronectin (α_5_β_1_, α_8_β_1_ α_v_β_1_, α_v_β_3_, α_v_β_5_, α_v_β_6_, α_v_β_8_, and α_II_β_3_).^246^^,^^247^^,^^248^ The binding of the respective integrin to the ECM initiates the process of mechanosensing and mechanotransduction, as reviewed by Sun and colleagues.^249^ The concept of cells sampling the stiffness of their environment by using a molecular clutch was first devised by Mitchinson and Kirschner.^250^ According to this hypothesis, the retrograde F-actin flow pulls on the ECM to which it is coupled via said molecular clutch consisting of integrins on the outside of the cell and adapter molecules (FAK, vinculin, talin,^251^ and paxillin^252^) on the inside of the cell^253^ (Figure 1). This generates cellular protrusions that mature from nascent adhesions via focal adhesions to fibrillar adhesions, being distinguished by their sizes, turnover times (ranging from minutes to hours), and molecular composition.^254^ Focal adhesions and their variants, therefore, convey information about increased stiffness into the cell. The molecular mechanism behind this is based on the fact that transient integrin and ligand bonds dissociate on soft substrates (slip bonds) without leading to enhancement of the bond by recruiting talin and vinculin. In contrast, on stiff substrates, this reinforcement occurs, leading to a more stable catch bond established through mechanical forces and further reinforced by vinculin and other molecules. Subsequent downstream signaling occurs mediated by other associated proteins such as FAK and triggers, on stiff surfaces, the nuclear translocation of YAP, which in turn leads to myofibroblast activation.^255^

In viscoelastic materials, the pull-induced deformation can also lead to a relaxation of the preexisting tension, leading to an abrogation of signaling. As reviewed by Chaudhuri and Mooney,^241^ studies using gels with a higher loss modulus, spreading, focal adhesion, and differentiation of stem cells were facilitated.^256^ In contrast, studies using fibroblasts demonstrated that those cells were unable to spread on soft elastic gels while they were forming stable focal adhesions and stress fibers, with evidence of YAP translocation on stiff elastic gels and, surprisingly, also on soft viscoelastic gels.^257^ Chaudhuri and colleagues theorized that this complex and unexpected behavior was based on the mechanics of how the motor clutch model can successfully sense stiffness on elastic 2D gels.^257^^,^^258^^,^^259^^,^^260^ Advanced computational methods were used to model this behavior and then validate it *in vitro.*^261^ In summary, this study found that when a gel is stiff, the bonds that connect the cell to the matrix are saturated, leaving viscous properties irrelevant to cell behavior. However, when gels are under a certain stiffness threshold (“soft”), cell spreading is ideal when the lifetime of the focal adhesion (T_L_) is longer than the substrate relaxation timescale (T_S_), which in turn is longer than the clutch-binding timescale (T_b_): The cell protrusions bind to the matrix and stay bound while it relaxes, leading to cell spreading.

### Durotaxis—Stiffness gradient-induced fibroblast migration

A seminal publication^262^ showed that dynamic pulling of focal adhesions leads to downstream activation of FAK with subsequent phosphorylation of paxillin on tyrosine 31 and 118, which leads to interaction between paxillin and vinculin. This mechanism was instrumental for the velocity of cells migrating along a stiffness differential in a process termed durotaxis,^263^ which has been demonstrated to occur *in vivo* in the embryonal development of Xenopus.^264^ In this process, the cytoskeleton reorganizes to form a protruding leading edge and a rear edge that is retracted to generate movement.^265^^,^^266^ Polarization of membrane tension seems to be relevant to the directionality of cell movement during this process,^267^ as well as a force imbalance that exists between different adhesion sites connecting with areas of different stiffness, and which is at least partly transduced via the nucleus.^268^ Of note, cells also show generally increased migration velocity on uniformly stiff surfaces within a certain range, probably because they can more efficiently “walk” over the matrix.^269^ Importantly, in this stiffness-driven migration, the stiffness gradient itself is the essential but not exclusive determinant of whether and how effectively cells migrate. Hartman and colleagues could demonstrate that vascular smooth muscle cells and fibroblasts were able to durotax on fibronectin-coated but not on laminin-coated gels with the same stiffness gradient, indicating that the ECM ligand composition determined how effectively the cells could respond to mechanical gradients.^270^^,^^271^ While it has been known for some time that fibronectin is relevant in the cellular response to ECM stiffness^272^^,^^273^ via binding to α5β1 to mediate FAK-c-Src activation,^274^ its role in cell migration is still the focus of ongoing research^275^ (Figure 1). Migrating cells lay out a fibronectin network that allows other cells to follow in their tracks when navigating 3D matrices via the same binding mechanism as their general stiffness-directed migration.^276^ In general, there is also evidence that migrating cells alter the ECM on their path to generate a spatial memory.^277^ A matrix-dependent mode of directed cell migration that needs to be distinguished from durotaxis is haptotaxis. Haptotaxis describes the migration of cells along a gradient of increasing ECM ligand density, such as fibronectin, by using lamellopodia.^278^ Beyond this, certain isoforms of fibronectin (fibronectin-containing extra type III domain A, FN-EDA) are produced at an increased rate in IPF fibroblasts and actively contribute to myofibroblast differentiation and propagation of IPF.^279^ Interestingly, it has been found that accumulation of FN-EDA in systemic sclerosis-associated interstitial lung disease (Ssc-ILD) fibroblasts is not based on increased transcription but on reduced turnover due to reduced ubiquitination,^280^ highlighting again that the matrix is not an inert deposition of proteins but a highly dynamic entity.

In summary (Figure 1), fibroblasts and other mesenchymal cells can be recruited to areas of increased stiffness via stiffness gradients and can be activated into myofibroblasts by this increased level of tissue stiffness.^281^^,^^282^ While it is compelling to speculate that fibroblasts are recruited to areas of increased stiffness (fibrotic foci) by durotaxis in IPF, whether durotaxis plays a decisive role in human fibrotic diseases *in vivo* is still an open question. Initial evidence exists for durotaxis of primary human lung fibroblasts;^283^ however, the most likely scenario integrates context-dependent durotaxis, haptotaxis, and chemotaxis.^284^^,^^285^

## Mechanosensing and intercellular force transmission

The transduction of mechanical force does not only occur from cell to matrix and vice versa, but also in between cells.^286^ Sunyer et al. could observe a collective durotaxis of epithelial cells on a fibronectin-coated gel with a stiffness gradient via long-distance force transmission through intercellular connections. There is evidence that cadherins are essential for sustained force transmission between cells in such collectively migrating monolayers.^287^ Recent results have suggested a sensing mechanism for intercellular force transduction similar to how cells sense the stiffness of the ECM, namely by pulling on the other cells. In this instance, transduction of force occurs via local contractions between E-cadherin cell-cell contacts facilitated by non-muscle myosin IIB and supported by the binding of vinculin and α-catenin.^288^ In fibroblasts, N-cadherins facilitate this intercellular mechanosensing via, among other mechanisms, stretch-sensitive calcium ion channels.^289^

Seminal work on asthma described the physical properties of a layer of human bronchial epithelial cells (HBECs) as fluid-like in an unjammed mobile phase that becomes jammed over time and then behaves more like a solid phase, a process that was delayed in asthmatic patients. In asthmatic HBECs, intercellular traction forces and tension were locally increased compared to controls but spanned shorter distances.^290^

Similar to this, a recent study found that bronchial epithelial cells from patients with IPF are pathologically unjammed and hypermobile.^291^ The authors could demonstrate that epidermal growth factor receptor (EGFR) activation and nuclear translocation of YAP were sufficient to cause epithelial unjamming in control cells. In turn, they successfully induced physiological jamming in these epithelial cells by blocking YAP translocation with verteporfin, a drug that inhibits YAP function by upregulating protein 14-3-3σ to sequester YAP in the cytosol, preventing its nuclear translocation.^291^^,^^292^ In co-culture experiments, exposure to unjammed epithelial cells (either naturally unjammed cells or pharmacologically unjammed control cells) led to the activation of human lung fibroblasts, which had been placed on soft gels to prevent direct mechanoactivation. Overall, this study provides compelling evidence that the initial epithelial mechanophenotype causes fibroblast activation via epithelial-mesenchymal crosstalk in IPF.^291^ In a follow-up study, the authors could additionally demonstrate that inhibition of integrin β_1_ or FAK signaling was able to cause jamming of pathologically unjammed epithelial cells.^293^

## Technical approaches to study the ECM and its physical properties

### Advanced matrix proteomics

In order to understand the role of the ECM and mechanobiological interactions between the matrix and cells, such as fibroblasts, technologies to measure and characterize all involved players are crucial. One approach to characterizing the ECM is the use of histology and immunofluorescence on lung tissue sections. This, however, is limited by the number of proteins and compounds that can be tested at once, and while it might be sufficient to study well-established molecules (e.g., fibronectin), it also limits researchers to already known targets for which antibodies are available. Advances in mass spectrometry-based proteomics^294^^,^^295^ have allowed for a more systematic and explorative approach regarding the ECM, generating an ECM protein atlas of murine organs, including network analyses.^296^ Furthermore, this approach has been used to comprehensively characterize human lung development^297^ and for the proteomic profiling of IPF lungs, which revealed proteins associated with disease severity.^298^ Another study was able to distinguish healthy individuals from IPF patients by applying a proteomics approach to plasma samples, also identifying a cohort of proteins associated with disease severity.^299^ A more recent large-scale proteomics study evaluated 2,921 proteins in plasma samples from an IPF patient cohort for discovery (*n* = 871) and then tested the findings on a validation cohort (*n* = 355) to successfully identify a signature of 140 proteins that were associated with three-year transplant-free survivals.^300^ Among them are potentially new disease-driving targets such as keratin 19. In conclusion, proteomic profiling could be used to predict disease progression and adapt the therapeutic strategy accordingly. Lastly, there is evidence for the promise of this approach beyond IPF, with a proteomics study using BAL of recovering COVID-19 patients to reveal persisting repair processes even nine months after clinically mild disease.^301^

With the advancement of spatially resolved omics technologies, mass spectrometry imaging (MSI)-based spatial proteomics has achieved the sensitivity to detect even the lowest amounts of proteins in the femtomolar range, as well as the spatial resolution to enable protein analysis with single-cell and even subcellular resolution.^302^ Some of these techniques are promising for single-cell resolution label-free high-throughput analyses like nanodroplet-processing in one pot for trace samples (nanoPOTS).^303^ Others, such as time-of-flight secondary ion mass spectrometry (ToF-SIMS), can be used to study proteomic interactions in subcellular resolution. Most spatial proteomics studies use laser capture microdissection in combination with mass spectrometry to characterize spatial compartments in the diseased lung.^304^ Using this approach, it was discovered that uninvolved fibrotic airway cells in IPF had a distinctively altered proteomic signature that was similar to that of airway cells involved in honeycomb formation.^305^ Highly sophisticated spatial proteomics studies using matrix-assisted laser desorption/ionization-MSI were able to generate a profile of the pulmonary metabolic landscape in murine disease models of lung fibrosis to reveal that lysosomal utilization of glycogen is a necessary feature of fibrotic lung disease.^306^ Another group used spatial metabolomics based on an air-flow-assisted desorption electrospray ionization-MSI system to characterize the metabolite distribution of an anti-fibrotic compound in lung fibrosis, thereby generating far-reaching insights into its mechanism of action by profiling metabolic changes, including tyrosine kinase phosphorylation, reactive oxygen species pathway activation, and ECM turnover.^307^ Spatial proteomic studies based on these techniques have the potential to complement other advanced imaging techniques, such as cytometry by time-of-flight, which is technically limited to “only” 50 different compounds at a time. Advanced proteomics, complementing spatial transcriptomics, also offers a promising perspective to better understand not only changes in the abundance of different ECM proteins but also posttranslational modifications and protein-protein interactions.

### Atomic force microscopy and nanoindentation

As the physical properties of the ECM are highly relevant in lung fibrosis, technologies to measure stiffness, viscoelasticity, and other properties are needed to complement, e.g., protein measurements. AFM is the current gold standard for measuring Young’s modulus and viscoelasticity of tissue.^308^^,^^309^ The setup works via a delicate cantilever that deviates a reflected laser beam, depending on the stiffness of the tissue it contacts, allowing for precise measurements on tissue sections *ex vivo* or in cell culture models. In experimental setups, AFM allows the mechanical characterization of pulmonary cell types such as fibroblasts, alveolar type II, and alveolar type I cells with subcellular resolution, as well as of decellularized ECM.^310^^,^^311^ AFM has also proven valuable in characterizing hydrogel-based *in vitro* assays that are instrumental in studying the effects of stiffness on fibroblasts and other cells.^312^ However, this technique comes with relevant limitations. As AFM measurements require direct sample contact and have only a small penetration depth, tissue measurements are only possible on explanted lung specimens. This poses limitations based on sample availability, as lung biopsies are performed less frequently to diagnose IPF, and only large transplant centers have access to a sizable number of explanted lungs. While AFM has an impressive spatial and stiffness/force resolution,^19^^,^^313^ allowing it to precisely characterize the surface stiffness of specific delicate structures such as the basement membrane^314^ or even models of surfactant film,^315^ it is not ideal for the measurement of larger tissue areas and volumes or even whole organs. Modern commercially available nanoindentation systems partially fill this gap, allowing the measurement of larger volumes, generating precise stiffness profiles of the overall tissue while requiring less complex setup and operation compared to classic AFM devices.^264^^,^^316^^,^^317^

### Brillouin, Raman, and second-harmonic generation

Brillouin microscopy is a contactless, non-invasive, label-free method that has recently been used to measure the stiffness of three-dimensional tissue samples and single cells with subcellular resolution.^318^ Brillouin microscopy assesses the stiffness of an object based on the frequency shift of a laser impulse that is sent into the sample. The laser impulse interacts and gets deflected by spontaneously occurring acoustic waves in the specimen, whose configuration is determined by the mechanical properties of the tissue. The interaction between laser impulse and acoustic waves results in a specific Brillouin frequency shift that allows conclusions about tissue stiffness.^319^^,^^320^^,^^321^ Recent technical advances have expanded its application to even extremely light-sensitive specimens^322^ and allow for scanning single cells,^323^ tissue,^324^ longitudinally developing organisms *in vivo,*^325^^,^^326^ and even subcellular mechanobiological responses with high temporal resolution.^320^ While there is currently no published data on the application of Brillouin microscopy in lung fibrosis research, promising data focusing on fibroblasts on a single-cell level,^322^^,^^327^^,^^328^ the ECM,^329^^,^^330^ and growing experiences with other organs^324^^,^^331^^,^^332^ show the promise of this technology to characterize the matrix and fibrotic tissue.

Raman spectroscopy, a method related to Brillouin microscopy, is based on the scattering of laser light by high-frequency optical phonons. It allows label-free quantification of biomolecules and is therefore useful in differentiating inflammatory from fibrotic processes in experimental lung diseases,^333^ especially when combined with other imaging techniques such as multiphoton microscopy, including second harmonic generation.^334^ Raman spectroscopy also allows insights into the composition of the ECM,^335^^,^^336^ identifying fibrosis via changes in label-free collagen imaging.^337^ Most practical applications are currently focused on fibrotic disease models,^338^ and while initial studies are underway to use Raman spectroscopy to diagnose IPF on frozen surgical specimens,^339^ most current studies focus on malignant pulmonary diseases.^340^^,^^341^

Second-harmonic generation (SHG) is a specific label-free and non-invasive technique using two-photon microscopy. SHG is based on the principle that two photons of the same wavelength, pulsed in quick succession, can interact to generate a new photon with half the wavelength. SHG can be used to visualize label-free several mechanobiology-relevant biomolecules, with microtubules,^342^ elastin,^343^ and collagen being the most visualized compounds in IPF studies.^344^ Complementarily, software methods are being developed to perform (semi-)automated quantitative structural analyses of fiber patterns found in the ECM.^345^

While each approach is promising in itself, approaches allowing for combined structural (2-photon microscopy, SHG) and stiffness imaging (Brillouin) promise a greater understanding of the interplay between matrix structure, its physical properties, fibroblasts, and other cells. Much remains unknown about the pathophysiology of fibrotic lung diseases, and, in some cases, a definitive diagnosis remains elusive despite histopathological analysis. Explorative studies aiming to identify ultrastructural changes, including collagen and elastin fiber orientation patterns in the matrix of IPF patients compared with controls and eventually to other fibrotic lung diseases, might contribute to improved classification and diagnosis in the future.^346^^,^^347^

### Real-time deformability cytometry

A highly innovative approach, real-time deformability cytometry (RT-DC), was pioneered by Jochen Guck and colleagues.^348^^,^^349^ RT-DC allows the high-throughput mechanical characterization of cells by using hydrodynamically induced deformation of the cells to compute Young’s modulus and other physical properties (e.g., cell size). This method is also combinable with fluorescence-based measurements of cell properties (e.g., FACS). Multiple studies have elucidated the mechanophenotype of immune cells,^350^ and recent advances in tissue processing have opened the way for mechanical phenotyping of primary human cells, such as fibroblasts, isolated from solid organs and biopsies.^351^ A recent RT-DC-based study successfully revealed increased stiffness, adherence to the ECM, and resilience to mechanical confinement in leucine-rich repeat-containing G-protein coupled receptor 5-positive colorectal cancer stem cells in patient-derived organoids.^352^ Initial studies in fibrotic lung disease show that patients with IPF have a distinctive neutrophil mechanophenotype with larger and stiffer neutrophils in IPF compared with controls with a positive correlation of neutrophil size and disease severity.^353^ This exciting and rapidly developing technique is particularly promising for high-throughput multi-omics approaches performing mechanical phenotyping in combination with other analytic methods, e.g., fluorescence-based protein expression analysis.

### Cross-sectional imaging modalities

Magnetic resonance elastography (MRE) has successfully been used to non-invasively quantify lung stiffness *in vivo* in spontaneously^354^ and even freely breathing patients^355^ by using magnetic resonance to detect the propagation of low-frequency shear waves in the lung. The waves are generated externally on the patient’s chest using vibration. MRE could show an average stiffness of 2.74 (±0.896) kPa in IPF patients compared to 1.33 (±0.195) kPa in healthy individuals.^356^ MRE was also able to distinguish patients with long COVID from healthy controls. Fascinatingly, repeated longitudinal MRE lung stiffness measurements in the long-COVID group over five months showed a decline of the initially increased stiffness, suggesting MRE could be used as a useful tool to monitor progression and response to therapy in fibrotic lung disease.^357^ In contrast to measuring fibrosis-inducing processes such as experimental single photon emission computed tomography (SPECT) of LOXL2,^358^ MRE actually measures an endpoint of disease manifestation, organ stiffness. To improve upon this basic concept, there have already been attempts to combine magnetic resonance imaging with collagen-specific diagnostic probes.^359^ Standalone non-invasive collagen imaging has also yielded promising initial results with contrast agent hProCA32 collagen-based magnetic resonance imaging in mice^360^ and tracer ^68^Ga-CBP8-based PET imaging in patients with IPF and healthy volunteers.^361^

## Mechanotherapeutic treatment approaches

### Current therapeutics

Traditionally, therapeutic approaches in IPF treatment have sought to prevent the progression of already existing fibrotic damage, as most patients are only diagnosed after considerable fibrosis is present and fibrotic tissue alterations are deemed irreversible. Pirfenidone, the first-in-class anti-fibrotic drug, has anti-inflammatory and anti-fibrotic properties, reducing the proliferation, migration, and collagen production of fibroblasts.^362^ It also reduces TGFβ signaling,^363^^,^^364^ thereby reducing collagen expression,^365^ ECM remodeling, and increase in stiffness.^366^ Also, pirfenidone reduces myofibroblast contractility by reducing αSMA expression and F-actin stress fiber formation.^367^ Transcriptome profiling also found a decrease in the elevated levels of the ECM protein cell migration-inducing and hyaluronan-binding protein under pirfenidone treatment, illustrating the effects of antifibrotic therapy on the composition of the ECM beyond collagen.^368^ Nintedanib is a multi-tyrosine kinase inhibitor, blocking, among others, fibroblast growth factor receptor 1, 2, 3, vascular endothelial growth factor receptor, and platelet-derived growth factor receptor α. Similar to pirfenidone, nintedanib seems to act on basic profibrotic pathways as it also shows effectiveness in fibrotic lung diseases other than IPF (e.g., SSc-ILD)^369^ by blocking TGFβ/SMAD2/3 signaling and ERK signaling.^370^

### Modulation of mechanosensing and mechanoactivation as a therapeutic concept

**Figure 2:**
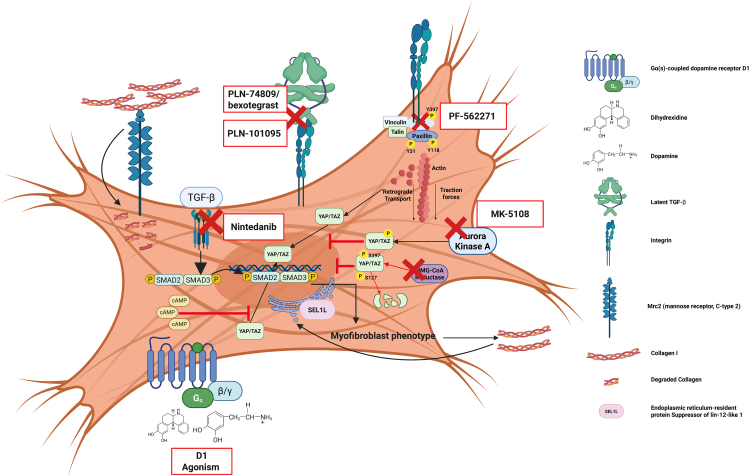
Mechanotherapeutic concepts Created in BioRender. Medoff, B. (2026) https://BioRender.com/h39ajhq. Potential targets for mechanotherapeutics. FDA-approved drug nintedanib blocks TGFβ-SMAD2/3 signaling, which reduces myofibroblast differentiation and indirectly reduces production of altered matrix. The experimental drug PLN-74809/bexotegrast (INTEGRIS-IPF; NCT04396756) directly blocks α_v_β_6_/α_v_β_1_ integrin-mediated activation of latent TGFβ, while the experimental drug PLN-101095 (NCT06270706) directly blocks α_V_β_8_/α_V_β_1_ integrin-mediated activation of latent TGFβ. Compound PF-562271 blocks FAK activation and reduces YAP translocation as a consequence in experimental murine models of lung fibrosis. Compound MK-5108 blocks aurora kinase A, which in turn leads to phosphorylation of YAP and its subsequent cytosolic retention. HMG-CoA reductase inhibitors, drugs also known as statins, are already FDA-approved for the treatment of hypercholesterolemia, increase phosphorylation of YAP on serine residues 397 and 127. This leads to cytosolic retention of YAP and its degradation, reducing mechanoactivation. Activation of the Gα(s)-coupled dopamine receptor D1 (DRD1), either by endogenous dopamine or the compound dihydrexidine, increases cAMP-dependent cytosolic retention of YAP in fibroblasts. The endocytic receptor Mrc2 (mannose receptor, C-type 2) is crucial for the endocytic uptake of collagen and its subsequent degradation. Upregulation of collagen synthesis in fibroblasts is registered by the endoplasmic reticulum-resident protein SEL1L and primes fibroblasts to later clear excessive collagen deposits, thereby resolving fibrotic remodeling of the matrix. SEL1L levels seem to be reduced in IPF.

Therapeutic interventions from a mechanobiology vantage point could, in principle, target the mechanical activation of latent TGFβ, the mechanoactivation of fibroblasts, the execution of mechanosensitive responses, and the fibrotic matrix itself Figure 2. As integrins play a crucial role in the mechanical activation of latent TGFβ and fibroblast mechanosensing, integrin-blocking therapies have been a target of interest in pulmonary fibrosis therapy for some time. Several publications, for example, have detailed the importance of integrins αvβ3 and αvβ5 for the myofibroblast phenotype in SSc fibroblasts by activating latent TGFβ in an autocrine fashion.^371^^,^^372^ Integrin blocking in IPF focuses on integrins overexpressed on local cell populations, such as epithelial cells and fibroblasts. Encouraging results suggest that the dual α_v_β_6_/α_v_β_1_ integrin inhibitor PLN-74809/bexotegrast successfully blocks latent TGFβ activation in a bleomycin model of lung fibrosis *in vivo* and in human PCLS *ex vivo*, resulting in reduced TGFβ pathway activation.^373^ PLN-74809/bexotegrast had recently entered clinical trials^374^^,^^375^ and had shown promising results in a multicenter phase IIa trial (INTEGRIS-IPF; NCT04396756) with favorable safety and tolerability endpoints as well as a reduced FVC decline over 12 weeks. The extent of reduced FVC decline was dose-dependent, and PLN-74809/bexotegrast had also reduced endpoints of fibrosis imaging. However, more recent data revealed that despite signs of efficacy early during the trial, patients on PLN-74809/bexotegrast did show an increased risk of IPF disease progression and toxicity later on, which led to discontinuation of the study.

Despite this setback, the therapeutic concept of blocking the mechanical activation of TGFβ through integrin inhibition is still being pursued. Supporting the fundamental role of mechanical TGFβ activation in fibrotic diseases, murine *in vivo* studies have indicated the effectiveness of PLN-74809/bexotegrast in a model of renal fibrosis based on ureter ligation.^376^ Furthermore, PLN-101095, an α_V_β_8_/α_V_β_1_-specific integrin inhibitor,^377^ is currently evaluated for cancer treatment in a phase I trial (NCT06270706). The aim of this development is to block TGFβ activation and its subsequent activity by means of integrin inhibition to disinhibit the anti-tumoral inflammatory response in patients with advanced metastatic solid tumors.

Against the backdrop of these mixed results, it remains to be seen if integrin inhibition will prove to be a viable therapeutic strategy in lung fibrosis. Assessing if a context-specific targeting of this mechanosensing pathway in the peritumoral stromal response might offer a more favorable cost-benefit ratio is one important avenue to pursue going forward. Beyond this, it needs to be emphasized that integrin-mediated mechanosensing is extremely complex. A recent study detailed how integrins α1β1, α2β1, and α11β1 facilitate collagen-binding-mediated fibroblast mechanoactivation, with their combined knockout alleviating fibrosis.^378^ Beyond this, integrin β1 also plays an important role in the activation of latent TGFβ.^379^ Hence, future research to better characterize the different facets of integrin-mediated mechanosensing is crucial to potentially uncover new vantage points to therapeutically modify it.

Inhibition of FAK to block fibroblast mechanoactivation with either PF-562271 or through siRNA successfully abrogated myofibroblast formation in a murine lung fibrosis model.^380^ In keeping with this, other authors found that blocking FAK activity led to reduced fibroblast activation and gene expression in lung fibroblasts and in the bleomycin-induced lung fibrosis model.^381^ However, current clinical trials evaluating FAK inhibition as a therapeutic concept have focused mostly on pulmonary malignancies.

One central step of mechanoactivation, nuclear translocation of YAP/TAZ in response to mechanosensing, has been specifically targeted and blocked by selective agonism of the Gα(s)-coupled dopamine receptor D1, which is predominantly expressed on mesenchymal cells in the lung. Blocking YAP/TAZ translocation in lung fibroblasts using this mechanism has been demonstrated to reverse their profibrotic phenotype and facilitate matrix degradation with stiffness reduction.^382^ Fascinatingly, Haak, Tschumperlin, and colleagues also found that DOPA decarboxylase, the enzyme concluding dopamine biosynthesis, was reduced in the lungs of IPF patients. Moreover, its expression levels were negatively correlated with disease severity, creating a potential mechanism to facilitate a targeted organ-specific D1 agonism as a therapeutic approach. Based on these results and given the centrality of YAP/TAZ in mechano-sensing and -activation of fibroblasts, pursuing YAP as a therapeutic target appears highly promising.^383^^,^^384^ In this vein, aurora kinase A has recently been identified as a new mediator in pulmonary fibrogenesis by using an exploratory screening approach. Targeting aurora kinase A with the specific inhibitor MK-5108 successfully blocked nuclear YAP translocation by inducing its phosphorylation-mediated cytosolic retention. In consequence, MK-5108 alleviated lung fibrosis in mice *in vivo.*^385^ There is also the potential to repurpose drugs already approved for other indications, with statins and the mevalonate pathway being identified as modulators of YAP translocation in fibroblasts in the same screening.^386^ Accordingly, HMG-CoA reductase inhibitors were capable of reducing fibrosis markers in both human fibroblasts and murine model systems of fibrosis by preventing an increased presence of YAP in the nucleus. Mechanistically, the drugs caused a relative increase in YAP phosphorylation on serine S127, which excluded YAP from the nucleus by facilitating binding to 14-3-3 proteins. Additionally, increased phosphorylation of serine 397 led to an increased YAP degradation. Another drug, verteporfin, which has FDA approval as a photosensitizer in the therapy of wet macular degeneration, has shown similar results, reducing YAP translocation in murine studies and *ex vivo* fibrosis models using PCLS through a similar mechanism.^387^ However, YAP also seems to have antifibrotic properties in epithelial cells,^388^ emphasizing the need for targeted modulation in fibroblasts and other mesenchymal cells.^389^ Despite these promising findings, there are currently no YAP inhibitors/modulators in advanced clinical trials for IPF.

### Remodeling of the fibrotic matrix as a therapeutic approach

With the goal of reversing fibrosis in mind, there have been efforts to therapeutically target the fibrotic ECM itself. Related to this, Yang and colleagues have designed a collagen-targeting liposomal vehicle. To deposit antifibrotic drugs, such as pirfenidone, specifically in locations with increased matrix for maximal effect, they used a collagen-binding targeting molecule with an attached collagenase to open up the fibrotic matrix for deeper deposition of the drug.^390^

A promising direct approach is to modulate collagen turnover to reduce, in a balanced and orderly fashion, the overall amount of collagen in the matrix.^391^^,^^392^ In addition to preventing the additional deposition of new collagen, effective remodeling of the ECM would also need to include the degradation of existing collagen. The extracellular pathway of collagen degradation requires the disintegration of cross-linked collagen fibers first. Matrisome-associated ECM regulators that play a vital role in this process are the MMPs,^393^ many of which are capable of degrading fibrillar collagen.^394^ Cathepsin K is another relevant collagen-degrading enzyme^395^^,^^396^ whose activity level was inversely correlated with the extent of ECM deposition in several pulmonary fibrosis studies.^397^^,^^398^ However, as McKleroy, Lee, and Atabai point out in their review,^391^ knocking out most MMPs does not actually lead to increased fibrosis, pointing to relevant dual functions for most of these enzymes and making direct targeting less useful. The exception in this regard is MMP-14, whose knockout leads to collagen accumulation. Mechanistically, the transmembrane MMP-14 is an important mediator of α_2_β_1_ integrin-mediated collagen phagocytosis and degradation.^399^

Importantly, the ECM in IPF is changed in a complex fashion with quantitative changes in multiple proteins and an altered ultrastructure, carrying the risk that simply targeting collagen for degradation could destabilize the resulting matrix, creating serious side effects. Consequently, “reprogramming” fibroblasts to remove collagen by comprehensively remodeling the matrix in a structured and orderly fashion seems the more holistic and sustainable approach. Mfge8 (milk fat globule epidermal growth factor 8), a core matrisome ECM glycoprotein, was found to be essential in limiting the fibrotic response in the murine bleomycin model. Mfge8-deficient mice had defective collagen turnover due to reduced collagen phagocytosis by macrophages, leading to collagen accumulation. Subsequently, these mice developed more pronounced lung fibrosis than controls.^400^ Similarly, an unbiased screen of *Drosophila* phagocytes identified cell division cycle 7 kinase as a negative regulator of the collagen endocytic receptor Endo180, making it a potential target in the mammalian system.^401^

Strikingly, it could be shown that cell-mediated collagen uptake and degradation are reduced in animals of old age, potentially due to impaired degradation of collagen as a consequence of downregulation of endocytosis receptors such as mannose receptor, C-type 2.^402^ This finding implies impaired matrix turnover in older individuals in response to injury as a potential explanation for why old age is a risk factor for developing IPF. In addition to understanding how cells degrade collagen intracellularly, a recent study successfully used a CRISPR screen to shine light on how cells sense that the degradation of collagen will become necessary.^403^ Fascinatingly, fibroblasts internally sense their own upregulation of collagen production via the endoplasmic reticulum-resident protein SEL1L (suppressor of Lin-12-like), which in turn primes the fibroblast for increased uptake and degradation of collagen. This mechanism, ensuring a homeostatic shutdown of the fibrotic injury response, seems to be impaired in IPF due to reduced SEL1L levels. Reprogramming these fibroblasts to counter their increased matrix production by reestablishing SEL1L function would potentially allow an induced therapeutic resolution of fibrosis.

## Conclusion

The ECM plays a crucial role in the development of lung fibrosis. Pulmonary fibroblasts produce a quantitatively and qualitatively altered matrix proteome (matrisome) that leads to spatially heterogeneous fibrosis with concomitant heterogeneous increases in stiffness. Fibroblasts possess the ability to sense these altered physical (stiffness) properties by probing their surrounding matrix as well as other cells (e.g., epithelial cells). They then integrate these profibrotic mechanical stimuli with other crucial pro-fibrotic signals such as TGFβ. In response, fibroblasts migrate toward areas of higher stiffness, differentiate into myofibroblasts, proliferate, and produce more altered matrix. Molecularly, mechanosensing is a complex process involving integrins and FAK-PI3K-Akt signaling among many other mechanosensors, with YAP translocation representing a central event in the mechanosensing cascade. In parallel with an improving understanding of mechanobiology, there have been tremendous technological advances supporting new discoveries, including spatially resolved advanced proteomics, contact-free optical methods such as Brillouin microscopy, and conceptually new approaches such as RT-DC. Building upon these mechanistic insights, new therapeutic concepts have emerged. Despite promising results in preclinical and early clinical trials, several “mechanotherapeutics” have recently failed in phase 2 and 3 clinical trials. While the anti-CCN2 antibody FG-3019/pamrevlumab did show a lack of effectiveness in the primary endpoint, the trial assessing the antibody PLN-74809/bexotegrast, which blocks mechanosensing integrins, was terminated due to a combination of increased risk of disease progression and toxicity. While these results represent significant setbacks, we still believe in the promise and general viability of mechanotherapeutics as a new class of drugs. For one, the developing and deepening understanding of the complex mechanobiology landscape leads to an ever-expanding list of promising targets, including but not limited to several sets of integrins, CCN2, FAK, dopamine receptors, aurora kinase A, and others. However, unknown redundancies and potential antifibrotic functions of molecules assumed to be solely profibrotic can contribute to unexpected negative outcomes of clinical trials. This is particularly relevant for the most promising approaches focusing on modulating fibroblast mechanosensing and reprogramming fibroblasts to directly remodel the fibrotic matrix. The latter approach has great potential as it could represent a truly regenerative approach by beneficially remodeling already established fibrosis. However, given its inherent complexity, we expect that additional rigorous basic and translational scientific studies will be necessary to achieve a more comprehensive understanding before a transition into clinical studies can occur. Despite these challenges, the quickly developing field of mechanobiology, carried by parallel technological advances allowing a better multidimensional (e.g., stiffness) characterization of biological systems, bodes well for the future. Last but not least, elucidating basic principles of mechanobiology also improves the chance that resulting “mechanotherapeutics” can be developed to target not only lung fibrosis but also other fibrotic diseases and even cancer.

## Acknowledgments

This work was supported by the 10.13039/501100001659Deutsche Forschungsgemeinschaft (German Research Foundation) through a Walter-Benjamin-Program Fellowship (project number 490745655) for I.G. This work was supported by 10.13039/100000002National Institutes of Health grants 5R01 HL157384 and 5R01 HL147059 to B.D.M. We gratefully acknowledge Professor Daniel J. Tschumperlin for his always insightful discussions on the topic of mechanobiology.

## Author contributions

I.G. conducted the literature research and wrote the original draft of the manuscript. B.D.M. provided critical revisions. Both authors contributed to the conceptualization and drafting of the review and approved the final manuscript.

## Declaration of interests

The authors declare no competing interests.
